# Thoracic epidural anaesthesia reduces insulin resistance and inflammatory response in experimental acute pancreatitis

**DOI:** 10.1080/03009734.2018.1539054

**Published:** 2018-11-23

**Authors:** Ola Winsö, Josef Kral, Wanzhong Wang, Ivana Kralova, Pernilla Abrahamsson, Göran Johansson, Per-Jonas Blind

**Affiliations:** a Department of Surgical and Perioperative Sciences, Anaesthesiology and Intensive Care Medicine, Umeå University, Umeå, Sweden;; b Department of Medical Biosciences, Pathology, Umeå University, Umeå, Sweden;; c Department of Surgical and Perioperative Sciences, Surgery, Umeå University, Umeå, Sweden

**Keywords:** Acute pancreatitis, epidural anaesthesia, insulin, microdialysis, sodium-taurocholic acid, sympathetic nervous system

## Abstract

**Aims:** The activity of the sympathetic nervous system (SNS) is crucial at an early stage in the development of an inflammatory reaction. A study of metabolic events globally and locally in the early phase of acute pancreatitis (AP), implying hampered SNS activity, is lacking.

We hypothesized that thoracic epidural anaesthesia (TEA) modulates the inflammatory response and alleviates the severity of AP in pigs.

**Material and methods:** The taurocholate (TC) group (*n* = 8) had only TC AP. The TC + TEA group (*n* = 8) had AP and TEA. A control group (*n* = 8) underwent all the preparations, without having AP or TEA. Metabolic changes in the pancreas were evaluated by microdialysis and by histopathological examination.

**Results:** The relative increase in serum lipase concentrations was more pronounced in the TC group than in TC + TEA and control groups. A decrease in relative tissue oxygen tension (PtiO_2_) levels occurred one hour later in the TC + TEA group than in the TC group. The maintenance of normoglycaemia in the TC group required a higher glucose infusion rate than in the TC + TEA group. The relative decrease in serum insulin concentrations was most pronounced in the TC + TEA group.

**Conclusion:** TEA attenuates the development of AP, as indicated by changes observed in haemodynamic parameters and by the easier maintenance of glucose homeostasis. Further, TEA was associated with attenuated insulin resistance and fewer local pathophysiological events.

## Introduction

Acute pancreatitis (AP) is a disease with two distinct phases, an early proinflammatory phase characterized by severe systemic inflammation and early multiple organ failure, followed by severe immunosuppression, leading to the development of infections and late multiple organ failure. AP carries a mortality rate ranging from 10% to 85% (1). The association in the early phase with a systemic inflammatory response implies both local and global metabolic changes, e.g. a change in insulin metabolism and liver injury (2,3). Plentiful challenges in the understanding of the pathophysiology of AP remain, one of which is the effect of blocking sympathetic nervous system (SNS) activity.

In clinical practice, thoracic epidural anaesthesia (TEA) can be used for pain relief in patients suffering from severe AP. A recent clinical study reports that TEA increases the arterial perfusion of the pancreas, thereby influencing the course of the condition favourably (4). Experimental studies are in agreement with this report (4), demonstrating the partial restoration of microcirculatory flow, thereby hampering the progression of histopathological changes, as well as systemic complications (2,5,6). In addition, SNS activity influences glucose and fat metabolism, by both direct neural and hormonal effects (7). Blocking SNS activity at the onset of AP could therefore possibly alleviate ensuing biochemical events, both globally and locally (8).

We designed an experimental set-up to study the effects of TEA in the early phase of sodium-taurocholate (TC)-induced AP, with the hypothesis that TEA would influence both global and local estimates of AP favourably. We examined whether haemodynamic parameters in animals with TEA would differ from those without. We furthermore evaluated metabolic changes occurring in the pancreatic parenchyma using the microdialysis technique and the histopathological examination of pancreatic specimens. We also assessed differences in global metabolic and inflammatory responses by determining glucose homeostasis and serum lipase concentrations with and without TEA respectively.

## Material and methods

The Animal Experimental Ethics Committee at Umeå University, Sweden, approved the study (reference number: A92-08), which was conducted in accordance with the Guide for the Care and Use of Laboratory Animals, National Research Council, Washington, USA, 1996. Twenty-five female pigs were used in the study. Their mean weight was 32.0 kg, ranging from 27 to 38 kg, i.e. juvenile pigs.

### Anaesthesia

The standard protocol for pig anaesthesia at our laboratory was followed. Intramuscular premedication using 10 mg/kg of ketamine (Ketalar^®^, Pfizer, Morris Plains, NJ, USA), 20 mg/kg of xylazine hydrochloride (Rompun vet, Bayer AB, Lyngby, Denmark), and 0.05 mg/kg of atropine sulphate (Atropin, NM Pharma, Stockholm, Sweden) was followed by the induction of anaesthesia by an intravenous bolus dose of 10 mg/kg of sodium pentobarbital (Pentobarbitalnatrium, Apoteksbolaget, Stockholm, Sweden). The animals were placed on a heating pad designed to maintain body temperature at 38 °C, as monitored continuously by a rectal thermometer. Anaesthesia was maintained by continuous infusions of 20 µg/kg/h of fentanyl (Fentanyl, Braun, Melsungen, Germany), 0.3 mg/kg/h of midazolam (Dormicum, Roche, Basel, Switzerland), and 5 mg/kg/h of sodium pentobarbital. For airway control, animals were tracheotomized and a 7.0 ID endotracheal tube was inserted (Rusch, Kernen, Germany). The pigs were normoventilated (Evita 4, Dräger, Kiel, Germany) using an air mixture containing 30% oxygen. Ventilation was gauged by intermittent arterial blood gas analyses (ABL 5 autoanalyzer, Radiometer, Copenhagen, Denmark), in combination with continuous end-tidal CO_2_ monitoring. Within the first hour, 1,000 mL of Ringer’s acetate was given, followed by a continuous infusion starting at 15 mL/kg/h. The infusion rate was adjusted to maintain central venous pressure (CVP) between 3 and 10 mmHg.

### Vascular access

Using a cut-down technique, a catheter was placed in the common carotid artery via a branch; a central venous line was inserted in the left external jugular vein, and a pulmonary artery (PA) catheter was inserted in the right external jugular vein. For pressure recordings, a catheter filled with isotonic saline and pressure transducers (DTX-pressure transducer, Becton Dickinson, Stockholm, Sweden) positioned at the mid-axillary level were utilized. The PA catheter was used for measurements of cardiac output (CO) using the conventional thermo-dilution technique and for continuous core temperature recordings. All the data were continuously recorded utilizing a computer-based multi-channel signal acquisition and analysis system (AcqKnowledge III, Biopac System Inc., CA, USA). Arterial blood was sampled for blood gas analyses.

### Epidural access

**Figure 1.**
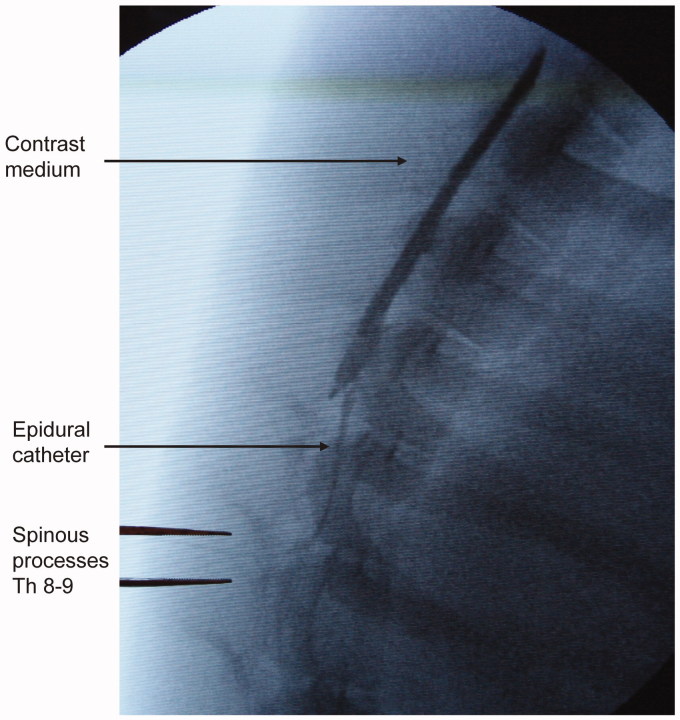
Initial phase of infusion of contrast medium into the epidural space.

The epidural space was accessed under radiographic guidance in the midline between the spinous processes of the eighth and ninth vertebrae, using a 20-gauge epidural needle (Braun, Melsungen, Germany) and the loss-of-resistance technique. The correct catheter position was verified by contrast-medium injection (Ultravist 300^®^; Schering AG, Berlin, Germany) via a standard epidural catheter in all animals (Figure 1). With the aim of blocking segments T5 to T12, the animals received 0.75 mL per segment of 0.5% bupivacaine (Marcaine, Astra Zeneca, Sweden) as a bolus dose, followed by 0.15 mL/kg/h as a continuous epidural infusion.

### Abdominal preparation

**Figure 2.**
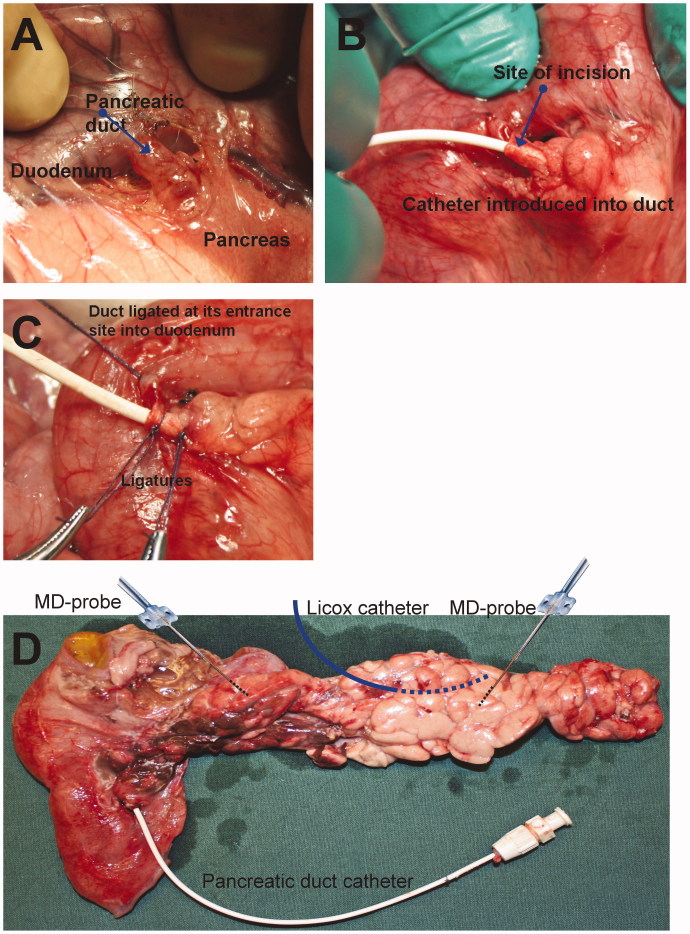
Panels A–C show the access, placement, and securing of a pancreatic duct catheter for taurocholate infusion. Panel D shows the placement of microdialysis probes, a Licox catheter, and a pancreatic duct catheter.

The abdomen was accessed by a midline laparotomy. A blood-flow recording ultrasound probe was placed around the portal vein (type 8SB, Transonic Systems Inc., USA). The pancreatic duct was identified at its entrance site into the duodenal wall (Figure 2A) by minimal dissection, as previously described in dogs (9). The duct was incised, and a 7 F catheter, with an olive at its distal end, was inserted a short distance into the duct, with the aim of minimizing preparatory trauma to the pancreas (Figure 2B). The catheter was retracted until its olive was stopped by a securing ligature (Figure 2C). The duct was also ligated at its entrance site into the duodenal wall.

**Figure 3.**
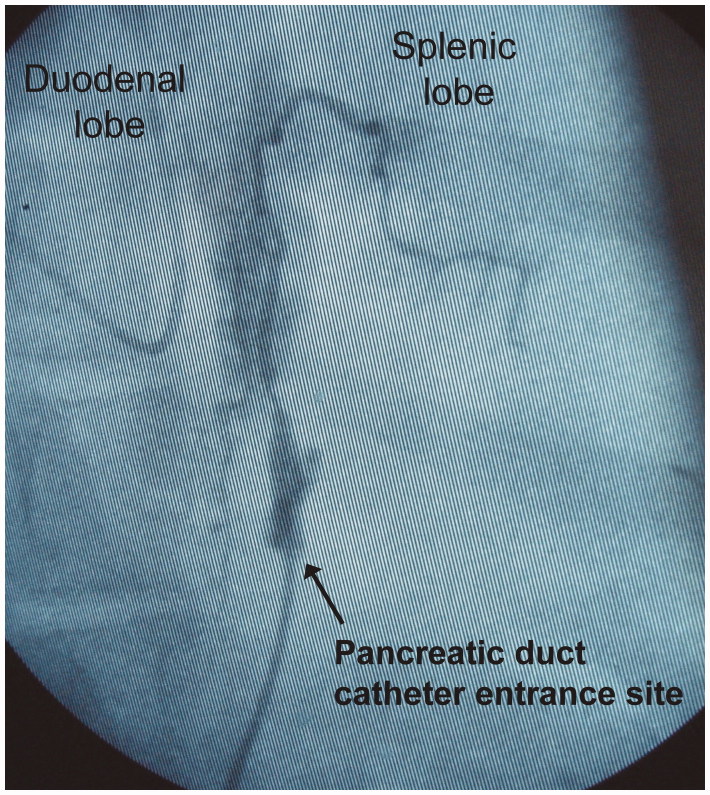
Infusion of contrast medium into the pancreatic duct. The contrast medium fills the entire length of the duct. In the duodenal lobe, acinary filling is observed. In the splenic lobe, the filling of first-generation duct branches is observed. No leakage of contrast medium at the catheter entrance site is observed. The volume of infused contrast medium corresponds to the volume of infused taurocholate.

The pancreatic duct catheter was externalized through a minimal right flank incision. When infusing contrast medium into the pancreatic duct, in the same way as infusing TC (see below), the total length of the main pancreatic duct was seemingly filled, as determined by fluoroscopy. There was no leakage of the infused contrast medium at the site of catheter entrance into the pancreatic duct, as observed by the naked eye, or as verified fluoroscopically. Acinar filling was obtained mainly in the duodenal lobe. The filling of at least first-generation ducts was obtained at the distal end of the splenic lobe (Figure 3). There was no sign of leakage of bile-stained fluid at the site of the pancreatic duct incision in any animal. Escape bile, gastric juice, and other duodenal contents could therefore be ruled out as contributing to the development of AP and a peripancreatic inflammatory reaction. Sutures used to secure the pancreatic duct catheter remained in place in all animals at the end of protocol time. The mode of limited pancreatic dissection involved the salvage of innervation, apart from along about 1 cm centred on the pancreatic duct entrance into the duodenal wall. We adopted the described technique of pancreatic handling for the following reasons. Firstly, the introduction of a catheter via a duodenotomy and the pancreatic papilla into the duct could jeopardize the reproducibility of TC infusion and thereby the method of inducing AP per se. If the tip of the catheter were placed in a wedged position in the duct, all the infused TC would be deposited upstream of the tip of the catheter, thus affecting only the corresponding part of the pancreatic parenchyma. Secondly, if the catheter were not placed in a wedged position, the infused proportion of TC actually deposited in the pancreatic ductal system, versus the proportion escaping retrogradely into the duodenum, would be impossible to determine.

A 12 F catheter was placed in the urinary bladder via a short supra-pubic midline incision, held in place using a purse string suture, followed by closing the incision.

### Microdialysis

Microdialysis probes with dialysis membranes of 10 mm in length allowing the passage of molecules up to 20 kDa were used (CMA 20, CMA Microdialysis, Solna, Sweden). One microdialysis probe was placed in the parenchyma of the duodenal lobe of the pancreas and one in the splenic lobe (Figure 2D) (10). The probes were fixed using a 5/0 suture onto the pancreatic capsula. All microdialysis probes were connected to a CMA 102 microdialysis pump (CMA Microdialysis, Solna, Sweden) and perfused by a modified Krebs-Ringer phosphate buffer solution (Fresenius Kabi, Halden, Norway). The perfusion rate was 1 µL/min. The dialysate sampling interval was 20 min throughout the experimental protocol. According to our laboratory practice, samples were collected in glass vials and sealed with crimp caps immediately after removal from the sampling tray (CMA/142 Microfraction collector, CMA Microdialysis, Solna, Sweden). Conforming with previously documented optimal handling, the samples were stored at 4–6 °C and centrifuged (Mini Galaxy, VWR, West Chester, PA, USA) for 30 seconds immediately before analysis within 24 h (11). Lactate and glucose were analysed in a CMA 600 analyser (CMA Microdialysis, Solna, Sweden). A tissue oxygen tension measurement probe (Licox CC1.2, Integra NeuroSciences, Plainsboro, NJ, USA) was inserted into the pancreatic parenchyma of the splenic lobe (Figure 2D). The shafts of the microdialysis probes were externalized by the midline incision.

Following completion of the preparation, the abdominal incision was immediately closed using a running suture, thereby minimizing heat loss and evaporation from the abdominal cavity.

Serum insulin was analysed using the ELISA technique (Mercodia Porcine Insulin ELISA, Mercodia AB, Global Headquarters, Sweden). Serum lipase was analysed using an enzymatic colorimetric method with 1.2-diglyceride in an Architect c4000 multianalyser (Abbott Laboratories, Abbott Park, IL, USA).

### Experimental protocol

One group of pigs (*n* = 8) had only TC-induced AP (TC group). Another group (*n* = 8) had TC-induced AP and epidural anaesthesia (TC + TEA group). A third group (*n* = 8), serving as controls for the evaluation of the effect of time, underwent all the preparations, apart from having pancreatic duct TC infusion or TEA. A fourth group would have been control + TEA, but it was not included, as this study focused on the effects of TEA on AP and not on the effects of TEA per se. Preparation was followed by a 60-minute equilibration period to achieve steady state. Two sets of baseline data were then obtained at 20-minute intervals. The last pig was used to validate the mode of delivery of TC into the pancreatic duct.

Infusions of TC into the pancreatic duct and bupivacaine into the epidural space were started simultaneously. The composition of the pancreatic duct infusion was 0.02 mol/L of CaCl_2_, 0.025 mol/L of Gly-Gly, 2 units/mL of trypsin, and 10% sodium taurocholate. The infusion was administered at a rate of 0.5 mL per kilo body weight during 20 min using an infusion pump. The animals in the control group received similar amounts of saline infused in the pancreatic duct. By extending the infusion time, we aimed to minimize any bursting effect by the volume of the infusion on the pancreatic ductal system. The mechanical bursting effect on the ductal system could be a contributory cause of inflammation by trauma, apart from the intended chemical one by TC. The effect of interventions on AP of dual aetiology would most probably have been more complicated to both assess and reproduce than AP with a single cause.

Samples were drawn from vascular catheters every 60 min throughout the total observation time (6 h). Aiming at a blood glucose concentration of 6 mmol/L, a continuous i.v. infusion of 5% glucose was administered and adjusted as needed. Blood glucose was measured every 60 min. At the end of the protocol period, the animals were sacrificed by an intravenous injection of a cocktail containing sodium pentobarbital and potassium chloride. Following cardiac arrest, the animals were exsanguinated into the thoracic cavity by dividing the inferior vena cava, prior to its entrance into the right atrium, with the aim of minimizing bleeding into the abdominal cavity when harvesting the pancreas. The total amount of intravenously administered glucose throughout the protocol period for each animal was calculated.

### Histopathological examination

Harvesting was started immediately after the animals were killed and completed within a few minutes. The pancreas was divided at the portal vein level, in the duodenal right and splenic lobes, and specimens approximately 5 × 5 × 5 mm in size were prepared. The specimens were immediately submerged in standard 4% formaldehyde solution in a 60 mL vial and marked with the running number of the pig and the lobe of the pancreas. From each vial, three to six specimens were placed in a cassette and embedded in paraffin. Five slides were prepared from each cassette and studied under the microscope. A point-scoring system listing eight parameters was designed. Each of the parameters—oedema in the pancreatic capsula, the migration of inflammatory cells into the capsula, the migration of inflammatory cells into septae between the acini, vasculitis in septal blood vessels, acinar inflammation estimated at less than 10%, 10%–50%, more than 50% of surface area on the slide, and necrosis—was assigned 1 (one) point. The maximum sum of points per specimen was therefore 8 (eight). Scores were calculated for the duodenal and splenic lobes separately. The pathologist was blinded to the origin of specimens.

### Statistical analysis

The data are presented as means and 95% confidence intervals. All data sets were tested for characteristics of distribution using Shapiro–Wilk’s test. No data set differed significantly from the normal distribution. The use of a paired *t* test was therefore justified. Repeated ANOVA was used to analyse variables over time. If the outcome of ANOVA was significant, comparisons versus baseline were made using a paired *t* test. For timewise longitudinal comparisons between compound concentrations at control and concentrations at each following time point for each individual probe position, a paired *t* test was used (intra-probe comparisons). For comparisons of compound concentrations across probe positions at each time point, Student’s *t* test was used (inter-probe comparisons). A *P* value of less than 0.05 was accepted as being indicative of a statistically significant difference between data sets. Fisher’s exact test was used to compute differences in proportions of points between the three experimental groups. The SPSS 22.0 software version (SPSS Inc., Chicago, IL, USA) was used for all analyses. A record in handwriting, available for audit, was kept for every animal during the course of the experimental procedure, according to laboratory regulations. Histological slides and all data files containing laboratory data are available for re-analysis, revision, and audit respectively. Furthermore, specimens stored in formaldehyde are available for further slide preparation and examination, if requested.

## Results

### Results pertaining to method of inducing pancreatitis

Macroscopically, all animals undergoing a TC infusion into the pancreatic duct had oedema and haemorrhagic areas in the duodenal lobe of the pancreas and oedema in the splenic lobe (Figure 2D), i.e. consistent with the appearance in previous experimental studies. To the naked eye, no difference in the degree of inflammatory reaction between the TC and TC + TEA groups was discernible. There were no macroscopic changes that could be assigned to AP in the control group, ruling out the experimental preparation as such, or the effect of time as causes of the findings in the two groups receiving TC.

### Haemodynamic parameters

The haemodynamic parameters are given in Table 1. Cardiac output (CO) decreased from 1 h and onwards in the TC group, but it did not change significantly in the TC + TEA group. The mean arterial pressure (MAP) decreased from 3 h and onwards in all three groups. The mean pulmonary arterial pressure (MPAP) increased from 1 h and onwards in the TC + TEA group and from 3 h and onwards in the TC group, but it did not change significantly in the control group. The systemic vascular resistance (SVR) decreased from 3 h and onwards in the TC + TEA group. The central venous pressure (CVP) increased from 3 h and onwards in the TC + TEA group. The pulmonary vascular resistance (PVR) increased from 3 h and onwards in the TC group.

**Table 1.** Hemodynamic parameters.

											ANOVA
			Baseline		1 h		3 h		6 h		within groups
	Group	*n*	mean	95% CI	mean	95% CI	mean	95% CI	mean	95% CI	*P*
CO	TC	8	4.9	±0.7	4.1	±0.7^a^	3.8	±0.6^a^	3.2	±0.8^a^	*<0.001*
	TC + TEA	8	3.7	±0.6	4.2	±1.0	3.8	±0.4	3.3	±0.7	*0.118*
	Control	8	3.8	±0.6	3.8	±0.5	3.1	±0.7^a^	3.1	±0.8	*0.037*
	*ANOVA*		*P = 0.004*		*P = 0.607*		*P = 0.117*		*P = 0.976*		
HR	TC	8	99.0	±16.8	86.4	±9.3	84.2	±11.8	88.1	±16.5	*0.121*
	TC + TEA	8	85.5	±10.0	87.8	±9.6	91.7	±12.7	85.4	±6.3	*0.260*
	Control	8	101.8	±17.5	109.8	±13.9	104.6	±15.6	92.5	±13.4	*0.073*
	*ANOVA*		*P = 0.156*		*P = 0.003*		*P = 0.058*		*P = 0.656*		
MAP	TC	8	91.9	±6.7	86.5	±15.9	76.8	±9.5^a^	68.4	±10.3^a^	*<0.001*
	TC + TEA	8	79.6	±4.2	73.9	±5.7	62.4	±7.4^a^	54.1	±7.5^a^	*<0.001*
	Control	8	95.5	±10.0	89.4	±8.9	74.5	±8.9^a^	69.5	±11.5^a^	*<0.001*
	*ANOVA*		*P = 0.002*		*P = 0.063*		*P = 0.023*		*P = 0.029*		
MPAP	TC	8	16.5	±1.7	16.4	±3.8	21.1	±3.2^a^	22.6	±2.6^a^	*0.003*
	TC + TEA	8	18.5	±3.7	21.8	±3.9^a^	22.6	±2.9^a^	25.4	±5.2^a^	*0.002*
	Control	8	21.6	±7.0	22.6	±4.7	23.1	±4.4	25.4	±7.1	*0.533*
	*ANOVA*		*P = 0.160*		*P = 0.042*		*P = 0.627*		*P = 0.593*		
CVP	TC	8	3.7	±3.8	3.0	±1.2	3.5	±1.5	3.8	±1.0	*0.495*
	TC + TEA	8	2.9	±1.1	3.8	±1.8	3.8	±1.2^a^	4.5	±1.6^a^	*0.032*
	Control	8	3.9	±2.7	2.9	±1.2	3.4	±1.3	5.4	±1.9	*0.003*
	*ANOVA*		*P = 0.605*		*P = 0.512*		*P = 0.837*		*P = 0.239*		
Q(port)	TC	8	859	±153	798	±243	728	±183	610	±165^a^	*0.023*
	TC + TEA	8	871	±217	803	±173	787	±143	657	±133	*0.239*
	Control	8	858	±184	763	±112	694	±190	845	±235	*0.103*
	*ANOVA*		*P = 0.991*		*P = 0.926*		*P = 0.669*		*P = 0.101*		
SVR	TC	8	18.23	±3.98	20.88	±3.93	20.02	±3.76	21.03	±2.84	*0.180*
	TC + TEA	8	21.58	±3.85	17.52	±3.44	15.46	±2.49^a^	16.13	±3.66^a^	*0.008*
	Control	7	23.98	±4.60	22.32	±3.81	24.48	±8.37	20.32	±5.23	*0.335*
	*ANOVA*		*P = 0.086*		*P = 0.111*		*P = 0.027*		*P = 0.090*		
PVR	TC	8	2.85	±0.75	3.44	±1.10	4.79	±1.08^a^	6.53	±2.32^a^	*0.002*
	TC + TEA	7	4.20	±1.29	4.38	±1.00	5.13	±1.05	7.21	±2.80^a^	*0.002*
	Control	7	4.50	±1.60	5.30	±1.63	7.04	±2.39	6.41	±1.42	*0.086*
	*ANOVA*		*P = 0.039*		*P = 0.081*		*P = 0.054*		*P = 0.818*		

Data are presented as mean ±95% confidence intervals. Repeated measures ANOVA within each group was used (absolute *P* values are shown) followed by paired *t* test versus baseline.

^a^*P* < 0.05 versus baseline. One-way ANOVA between the three groups was also used, and absolute *P* values are shown.

CO = cardiac output (L/min); CVP = central venous pressure (mmHg); HR = heart rate (bpm); MAP = mean arterial pressure (mmHg); MPAP = mean pulmonary arterial pressure (mmHg); PVR = pulmonary vascular resistance (mmHg/[L/min]); Q(port) = portal venous blood flow (mL/min); SVR = systemic vascular resistance (mmHg/[L/min]).

### Lipase in serum

**Figure 4.**
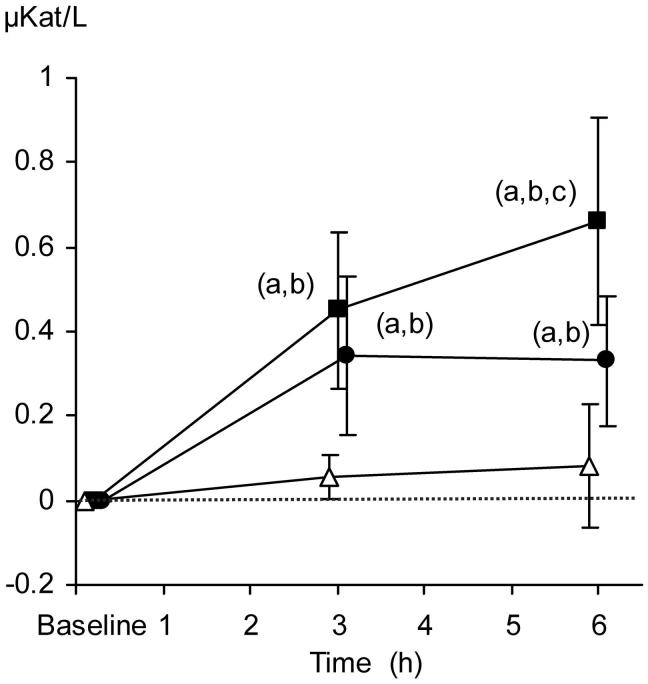
Relative changes in serum lipase concentrations (µkat/L). The data are presented as the mean ±95% confidence intervals. Control group (open triangles), TC group (filled squares), and TC + TEA group (filled circles). For all groups, *n* = 8. (a) *P* < 0.05 using repeated measures ANOVA within groups followed by a paired *t* test versus baseline values. One-way ANOVA was used to compare the three groups, followed by an independent-samples *t* test. (b) *P* < 0.05 versus control group. (c) *P* < 0.05 versus TEA group.

From baseline to the end of the protocol period, median lipase concentrations increased from 0.106 to 0.188, from 0.144 to 0.475, and from 0.013 to 0.675 µkat/L in the control group, TC + TEA, and TC groups, respectively. The relative increase in serum lipase concentrations was more pronounced in the TC group than in TC + TEA and control group at 6 h (Figure 4).

### Tissue oxygen tension

#### Comparison over time.

**Figure 5.**
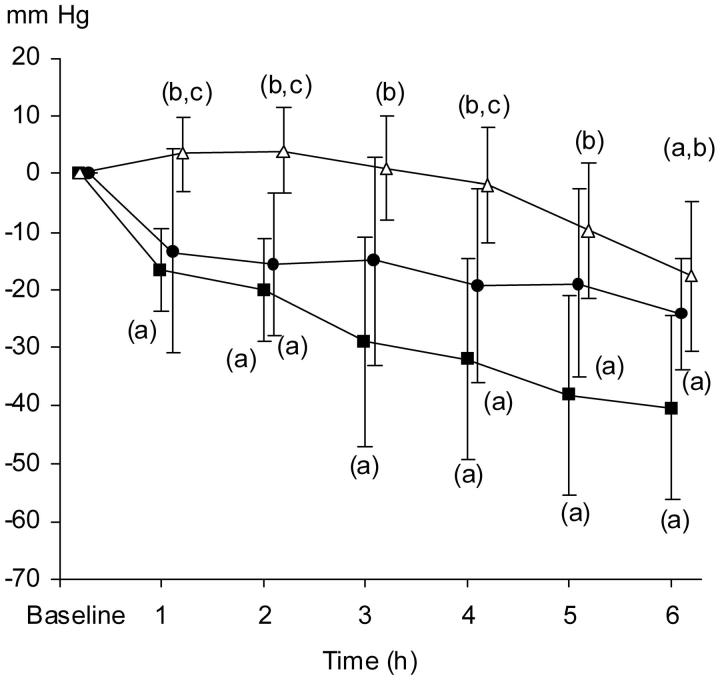
Relative changes in pO_2_ tissue (mmHg) in pancreatic parenchyma. The data are presented as the mean ±95% confidence intervals. Control group (open triangles, *n* = 8), TC group (filled squares, *n* = 8), and TC + TEA group (filled circles, *n* = 6). (a) *P* < 0.05 using repeated measures ANOVA within groups, followed by a paired *t* test versus baseline values. One-way ANOVA was used to compare the three groups, followed by an independent-samples *t* test. (b) *P* < 0.05 versus TC. (c) *P* < 0.05 versus TEA group.

The relative tissue oxygen tension (PtiO_2_) decreased continuously during the protocol period in the TC and TC + TEA groups as compared to baseline. The course of parenchymatous oxygen tension in the pancreas, in animals with TC, was thus consistent with that of progressive oedema formation, ensuing hampered capillary blood flow, and inflammation (Figure 5).

#### Comparison between groups.

Relative PtiO_2_ levels were lower in the TC group than in the control group from 1 h and onwards. Relative PtiO_2_ levels were lower in the TC + TEA group than in the control group from 2 h and onwards, thus occurring 1 h later than in the TC group. In the control group, the relative PtiO_2_ only decreased at 6 h, probably depending on preparatory trauma (Figure 5).

### Glucose infusion rate

**Figure 6.**
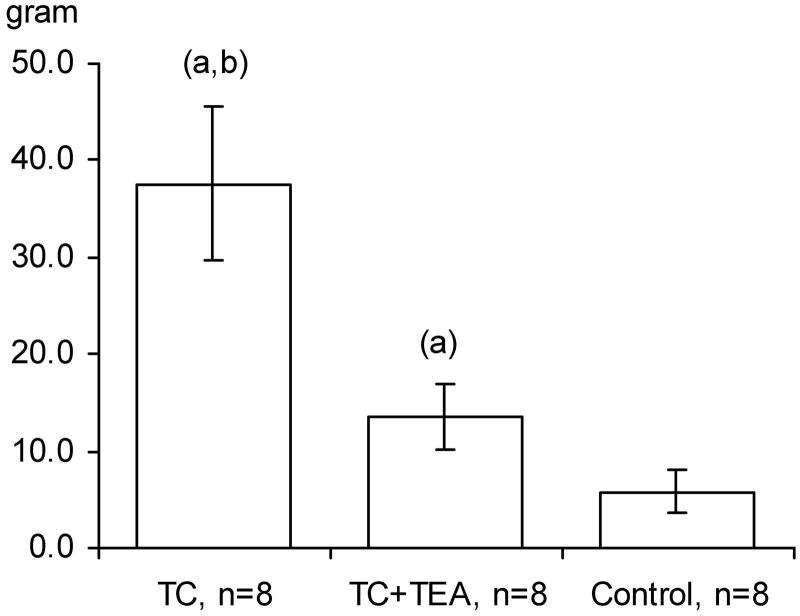
Total amount of 5% glucose administered to maintain normoglycemia in the TC group, TC + TEA group, and control group (for all groups, *n* = 8). The data are presented as the mean ±95% confidence intervals. An independent-samples *t* test between groups was used to compare the total amount of glucose administered. (a) *P* < 0.05 versus control. (b) *P* < 0.05 versus TEA.

The maintenance of normoglycaemia (glucose concentration of 6 mmol/L in arterial blood) in the control group was attained at a steady intravenous infusion rate of 5% glucose throughout the protocol period. In the TC + TEA group, a higher steady infusion rate was needed to maintain the predetermined glucose level. Strikingly, the accumulated amount of glucose administered for the maintenance of normoglycaemia was higher in the TC group than in the TC + TEA group, as well as significantly higher in the TC + TEA group versus the control group (Figure 6). Interestingly, the maintenance of normoglycaemia in the TC group required a higher, continuously increasing glucose infusion rate from 3 to 5 h in the protocol period than in the TC + TEA group (detailed data not shown).

### Serum insulin concentrations

**Figure 7.**
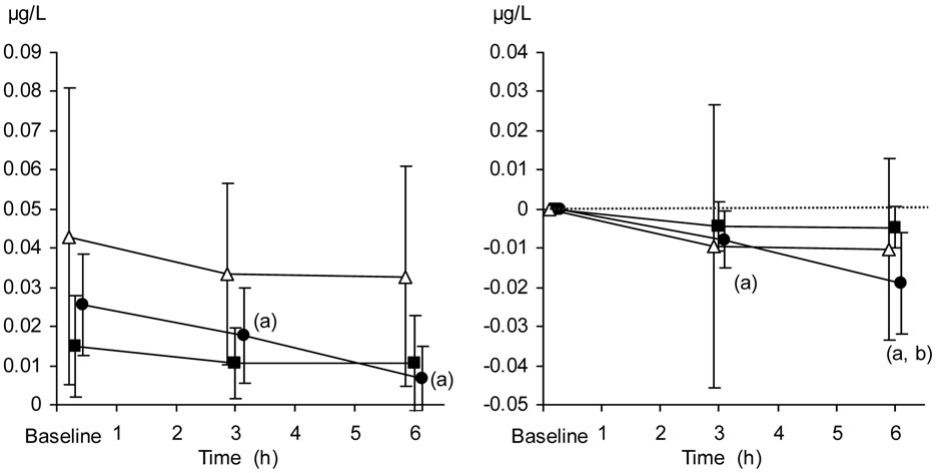
Serum insulin concentration (µg/mL). Absolute values (left panel) and relative changes from baseline (right panel). The data are presented as the mean ±95% confidence intervals. Control group (open triangles), TC group (filled squares), and TC + TEA group (filled circles). For all groups, *n* = 8. (a) *P* < 0.05 using repeated measures ANOVA within groups, followed by a paired *t* test versus baseline values. (b) *P* < 0.05 TC + TEA group versus TC group using an independent-samples *t* test to compare groups at 6 h.

The relative decrease in serum insulin concentrations was significantly more pronounced in the TC + TEA group than in both the TC and the control group at 6 h, as compared to baseline. Absolute concentrations of insulin, as well as relative changes, are presented in Figure 7.

### Glucose in pancreatic parenchymal microdialysis dialysate

#### Duodenal lobe of the pancreas.

As compared to baseline, the decrease in glucose concentration was more pronounced in the TC group (from 1 h and onwards) than in the TC + TEA group (at 6 h) (Table 2).

**Table 2.** Microdialysis concentrations of glucose and lactate in pancreatic parenchyma.

											ANOVA
			Baseline		1 h		3 h		6 h		within groups
	Group	*n*	mean	95% CI	mean	95% CI	mean	95% CI	mean	95% CI	*P*
Pancreas duodenal lobe											
GLU	TC	8	2.87	±0.64	2.30	±0.66^a^	1.24	±0.55^a^	0.57	±0.48^a^	*<0.001*
	TC + TEA	7	1.97	±0.79	1.52	±0.75	0.88	±0.94	0.61	±0.68^a^	*0.044*
	Control	6	2.37	±0.91	2.40	±0.95	2.14	±0.76	1.76	±0.87	*0.100*
	*ANOVA*		*P = 0.133*		*P = 0.126*		*P = 0.033*		*P = 0.003*		
LAC	TC	7	1.06	±0.29	1.24	±0.29^a^	1.47	±0.36^a^	1.73	±0.84^a^	*0.037*
	TC + TEA	7	1.05	±0.25	1.21	±0.25	1.23	±0.58	1.50	±0.65	*0.129*
	Control	6	1.13	±0.24	1.21	±0.20	1.36	±0.22^a^	1.54	±0.24^a^	*0.013*
	*ANOVA*		*P = 0.880*		*P = 0.965*		*P = 0.600*		*P = 0.752*		
Pancreas splenic lobe											
GLU	TC	8	2.32	±0.60	2.26	±0.42	1.19	±0.49^a^	1.25	±0.65^a^	*0.001*
	TC + TEA	7	2.52	±1.25	2.19	±0.88	1.42	±0.94	0.88	±0.80	*0.063*
	Control	6	1.90	±1.45	1.77	±1.56	1.31	±1.34	0.91	±1.00^a^	*0.047*
	*ANOVA*		*P = 0.630*		*P = 0.636*		*P = 0.905*		*P = 0.657*		
LAC	TC	8	0.90	±0.32	1.12	±0.39^a^	1.37	±0.57^a^	1.54	±0.64^a^	*0.006*
	TC + TEA	7	1.19	±0.31	1.31	±0.32	1.41	±0.38	1.81	±0.53^a^	*0.022*
	Control	7	1.22	±0.26	1.38	±0.32^a^	1.59	±0.42^a^	1.62	±0.72^a^	*0.034*
	*ANOVA*		*P = 0.155*		*P = 0.437*		*P = 0.704*		*P = 0.736*		

Data are presented as mean ±95% confidence intervals. Repeated measures ANOVA within each group was used (absolute *P* values are shown) followed by paired *t* test versus baseline.

^a^*P* < 0.05 versus baseline. One-way ANOVA between the three groups was also used, and absolute *P* values are shown.

GLU = glucose (mmol/L); LAC = lactate (mmol/L) in pancreas head and pancreas tail.

#### Splenic lobe of the pancreas.

As compared to baseline, the decrease in glucose concentrations in the TC group reached significance from 3 h and onwards. In the TC + TEA group, no significant changes were recorded (Table 2).

There were no significant differences in the glucose concentrations in pancreatic parenchymal dialysate between the duodenal and splenic lobe (Table 2).

### Lactate in pancreatic parenchymal microdialysis dialysate

#### Duodenal lobe of the pancreas.

As compared to baseline, lactate concentrations in the pancreatic parenchymal microdialysis dialysate in the TC group increased continuously, reaching statistical significance, as compared to baseline levels, just 1 h into the protocol period (Table 2). In contrast, in the TC + TEA group, lactate concentrations did not change significantly during the entire protocol period. Surprisingly, the lactate concentrations in the control group increased significantly from 3 h and onwards, as compared to baseline.

#### Splenic lobe of the pancreas.

As compared to baseline, the lactate concentrations in the pancreatic parenchymal microdialysis dialysate in the TC group increased continuously, reaching statistical significance just 1 h into the protocol (Table 2). As compared to baseline, the elevation of lactate concentrations in the TC + TEA group only reached significance at 6 h into the protocol period. Surprisingly, the lactate concentrations in the control group increased significantly from just 1 h and onwards, as compared to baseline (Table 2).

### Histopathological examination

Histopathologically, the appearance of the pancreas in all animals receiving taurocholate was consistent with that of AP. There was no significant difference in the sum of points between the TC + TEA and TC groups. As expected, the sum of points differed between both groups receiving TC and the control. Interestingly, from a methodological point of view, in both groups receiving TC, the sum of points was higher in the duodenal lobe than in the splenic lobe of the pancreas (data not shown).

## Discussion

Our findings demonstrate that establishing TEA, when inducing AP, attenuates the development of AP, as indicated by the observed changes in haemodynamic parameters and by the easier maintenance of glucose homeostasis. Further, TEA was associated with attenuated insulin resistance and fewer local pathophysiological events.

### Global effects

The global beneficial effects of blocking SNS by TEA are demonstrated by the course of haemodynamic parameters being less altered in animals with TEA than in those without. A further beneficial effect of suppressing SNS is manifested globally by lower concentrations of lipase in the bloodstream in animals with TEA, compared with those without. An effect of this kind could depend on differences in integrity in the acinar cells, the transport of the enzyme from the pancreas into the blood stream, and the clearance of the enzyme from the blood in the respective groups.

As reported, in experimental set-ups, juvenile pigs are prone to hypoglycaemia. They consequently require a continuous i.v. infusion of glucose to prevent hypoglycaemia (12). In man, hypoglycaemia is regarded as an indicator of an overwhelming inflammatory response (13,14). The need for an increased continuous glucose infusion in an attempt to maintain normoglycaemia in our experimental model is therefore interpreted as an indicator of developing a severe pancreatic inflammation.

To the best of our knowledge, the dramatic reduction in the need for intravenous glucose administration to maintain normoglycaemia in animals with TEA, as compared to those without, has not previously been reported. This finding is all the more striking when serum insulin courses are compared between the groups. TEA seemingly has a favourable effect on insulin homeostasis. An effect of this kind is synergistic with the endocrine function of the pancreas, apparently withstanding AP more effectively, than the exocrine one. In fact, it has been reported that the complete failure of exocrine pancreatic function may coexist with normal endocrine function in patients suffering from AP following pancreatic transplantation (15). Apart from an intra-pancreatic impact on glucose homeostasis, we speculate that TEA could possibly diminish peripheral insulin resistance, thereby reducing the need for insulin secretion. A global effect of this type is in agreement with the reported alleviation by TEA of liver, lung, and intestinal injury in pancreatitic rats (16).

### Intra-pancreatic effects

Our data indicate that TEA possibly has a protective effect on acinar, ductal, and endothelial cell integrity, comprising oedema formation, as manifested by a difference in the course of tissue oxygen tension between the TC + TEA and TC groups. Lactate concentrations in dialysate collected by intra-parenchymatously placed microdialysis probes correlate well with intra-pancreatic tissue oxygen tension in an experimental pancreatic ischemia-reperfusion model in pigs (17). Lactate concentrations thus mirror the intra-pancreatic metabolic status with a high degree of accuracy. TEA seemingly has beneficial effects when considering intra-pancreatic metabolic events, as judged by the course of lactate and glucose concentrations in retrieved extracellular dialysate. The aerobic metabolism in the pancreatic parenchyma is in fact maintained for longer in the TC + TEA versus the TC group of animals. It has been reported that TEA partially restores microcirculatory flow in the pancreas, as gauged by laser-Doppler flowmetry, apart from impeding inflammatory progression from oedematous to necrotizing pancreatitis (5).

These observations could be explained, firstly, by the suppression of the SNS effect by TEA. In patients undergoing surgical trauma associated with total knee arthroplasty, the elevation of biochemical markers associated with a general inflammatory response is reported to be less pronounced in those having lumbar epidural anaesthesia followed by epidural analgesia than in those having spinal anaesthesia followed by i.v. morphine analgesia (18). Secondly, the beneficial effect of TEA could be a general effect of the anaesthetic escaping from the epidural space into the global circulation, i.e. exerting a global anaesthetic effect. In fact, bupivacaine has an anti-inflammatory effect in ultralow concentrations in an *in-vitro* experimental set-up (19,20). It has been reported that only a very small fraction of administered local anaesthetic accounts for the anaesthetic effect, while most of it is absorbed into tissues or the systemic circulation (21). The effect of TEA could, furthermore, be a beneficial synergetic effect of the explanations suggested above. Observations in the control groups in our experimental set-up strongly contradict the effect of time as an explanation of these observations.

From the AP-inducing methodological point of view, it is noteworthy that, in animals receiving TC, the inflammatory reaction, also gauged histopathologically, was more pronounced in the duodenal lobe than in the splenic lobe. Had the method of inducing AP implied extensive acinary filling in the total length of the pancreas, this difference would probably not have emerged. Our observation apparently contradicts overfilling, i.e. bursting of the pancreatic acinary systems, as a contributory cause of AP. Our method of inducing AP is therefore mainly chemical, although a mechanical pressure effect cannot be ruled out.

### General considerations

In this study, TEA and the infusion of TC were started simultaneously. The idea for this study emanates from the clinical observation that patients with acute pancreatitis treated with TEA often do surprisingly well. Whether TEA established at some time point following the induction of AP would be beneficial cannot be determined from the present study and remains to be further investigated. It seems reasonable, however, to speculate that some effect of TEA, established in this way, would be present. A lack of reports on the occurrence of AP in patients with already established TEA could be interpreted as meaning that TEA does in fact have a protective effect on triggering AP. Furthermore, clinical reports of unfavourable effects of TEA on the course of AP are lacking.

To conclude, based on both global and local estimates, our findings indicate that the administration of bupivacaine into the epidural space has a favourable effect on the course of AP. Globally, TEA dramatically reduces the need for glucose administration in order to maintain normoglycaemia and has a favourable effect on haemodynamic estimates. Locally, TEA reduces enzyme leakage and affects cellular metabolism favourably, i.e. apparently preserving pancreatic cellular integrity. Our findings in relation to the effect of TEA on the course of AP support the concept of a neuro-immune property counteracting inflammation.
